# Permethrin as a Potential Furin Inhibitor through a Novel Non-Competitive Allosteric Inhibition

**DOI:** 10.3390/molecules28041883

**Published:** 2023-02-16

**Authors:** Dongyan Feng, Le Ren, Jiaqi Wu, Lingling Guo, Zhitao Han, Jingjing Yang, Wei Xie, Yanbing Wang, Fanxing Xu, Xin Su, Dahong Li, Hao Cao

**Affiliations:** 1 School of Life Science and Biopharmaceutics, Shenyang Pharmaceutical University, 103 Wenhua Road, Shenyang 110016, China; 2 Microbial Research Institute of Liaoning Province, Chaoyang 122000, China; 3 School of Integrated Chinese and Western Medicine, Nanjing University of Chinese Medicine, Nanjing 210023, China; 4 Wuya College of Innovation, Shenyang Pharmaceutical University, 103 Wenhua Road, Shenyang 110016, China; 5 School of Traditional Chinese Materia Medica, Shenyang Pharmaceutical University, 103 Wenhua Road, Shenyang 110016, China

**Keywords:** furin inhibitor, pyrethrin I, permethrin, non-competitive inhibitor, allosteric inhibitor

## Abstract

Furin is a potential target protein associated with numerous diseases; especially closely related to tumors and multiple viral infections including SARS-CoV-2. Most of the existing efficient furin inhibitors adopt a substrate analogous structure, and other types of small molecule inhibitors need to be discovered urgently. In this study, a high-throughput screening combining virtual and physical screening of natural product libraries was performed, coupled with experimental validation and preliminary mechanistic assays at the molecular level, cellular level, and molecular simulation. A novel furin inhibitor, permethrin, which is a derivative from pyrethrin I generated by *Pyrethrum cinerariifolium* Trev. was identified, and this study confirmed that it binds to a novel allosteric pocket of furin through non-competitive inhibition. It exhibits a very favorable protease-selective inhibition and good cellular activity and specificity. In summary, permethrin shows a new parent nucleus with a new mode of inhibition. It could be used as a highly promising lead compound against furin for targeting related tumors and various resistant viral infections, including SARS-CoV-2.

## 1. Introduction

Furin was identified via a comparative analysis of the sequence encoding the upstream region of the FES/FPS proto-oncogene [1,2]. It is one of the most important protein convertases; as a Ca^2+^-dependent serine endoprotease [3] belonging to the *Bacillus subtilis* protease-like protease family, is involved in the maturation of many secreted proteins and plays an important role in normal physiological activities [4].

Furin is associated with a variety of diseases, such as cardiovascular diseases and endocrine system disorders, and is especially closely related to viral infections and tumors [5,6,7,8]. With the outbreak of COVID-19 in the last three years, research related to furin-mediated viral infections has been accelerated. The SARS-CoV-2 virus infects humans mainly via the spike protein [9,10,11,12], and priming of the spike protein is known to be a necessary step. One of the major evolutionary steps in spike of SARS-CoV-2 over SARS has been demonstrated by researchers, including us, to be the evolution of furin-like enzymatic cleavage sites at the S1/S2 loci [10,13], which makes it more susceptible to hydrolysis by TMPRSS2 and furin, also increasing its ability to infect and spread [14,15]. Inhibition of furin activity reduces the probability of spike protein cleavage and thus blocks the pathway of SARS-CoV-2 into the host cell [10,15]. On the other hand, many studies have shown that furin is highly expressed in a variety of tumor tissues and cells, and that inhibition of furin can inhibit the tumorigenicity of colon, liver, and rectal cancer cells in vitro and/or in vivo [16,17]. In addition, several clinical trials have demonstrated the therapeutic efficacy of autologous vaccines against furin in cancer patients with different types of tumors [18,19]. Therefore, the development of inhibitors for furin, as an important target for various diseases, is a crucial challenge. However, most of the existing inhibitors are based on furin’s suitable substrate peptides, such as α1-antitrypsin variants, meta-guanidinomethyl-Phac-RVR-Amba, RVKR-CMK, etc., [20,21,22,23]; they have great inhibitory capacity but lack structural diversity and novelty, which can lead to the development of drug resistance. More potential inhibitors with novel structures need to be discovered to enrich the types of furin inhibitors and for structural–functional studies of furin. Notably, natural products are an important source of drug discovery, especially for antiviral and antitumor compounds and drug candidates. Meanwhile, with the rapid development of structural biology, molecular biology, and computer science, as well as the continuous advancement of high-throughput screening techniques, target-based drug discovery strategies have been widely used.

In this study, on the one hand, over 200,000 compounds were virtually screened using computer-aided drug design. On the other hand, inhibition of furin protein with high purity was analyzed by physical screening on 3000 compounds of natural products selected after virtual filters. Several compounds with furin inhibitory activity were obtained. Finally, permethrin was the most important compound found in this study, which has not been reported to have inhibitory activity against furin. Permethrin is a pyrethrin I derivative, which is the main active component of *Pyrethrum cinerariifolium* Trev. Permethrin is the most commonly used synthetic pyrethroid insecticide and is widely used due to its high activity as an insecticide and low mammalian toxicity [24], meanwhile, it is also used as a potential therapeutic compound for diseases such as scabies, Ofuji disease, and breast cancer [25,26,27].

In addition to the selective inhibitory ability of permethrin on furin and its high activity at the cellular level, this research has found for the first time that permethrin can be used as a new type of inhibitor targeting the allosteric pocket of furin through enzymatic kinetic studies combined with extensive theoretical calculations, which will help us to better understand the relationship between the structure and function of furin. At the same time, this novel form of allosteric non-competitive inhibition will also provide inspiration for the development of novel inhibitors of furin.

## 2. Results

### 2.1. Expression, Purification, and Activity Assay of Furin

Expression of furin was identified and purification was achieved using SDS–PAGE and Western blot results with a target band at 52 KDa (Figure S1A–C). Boc-R-V-R-R-AMC [28] can be specifically cleaved by furin to dissociate AMC, which can be detected using a fluorescence spectrophotometer with 370 nm excitation and 460 nm emission wave lengths. The enzyme activity was calculated after the assay. The results showed that the furin obtained in this research has a high activity and could be used for further studies (Figure S1D).

### 2.2. Virtual and Physical Screening of Furin Inhibitors

**Figure 1:**
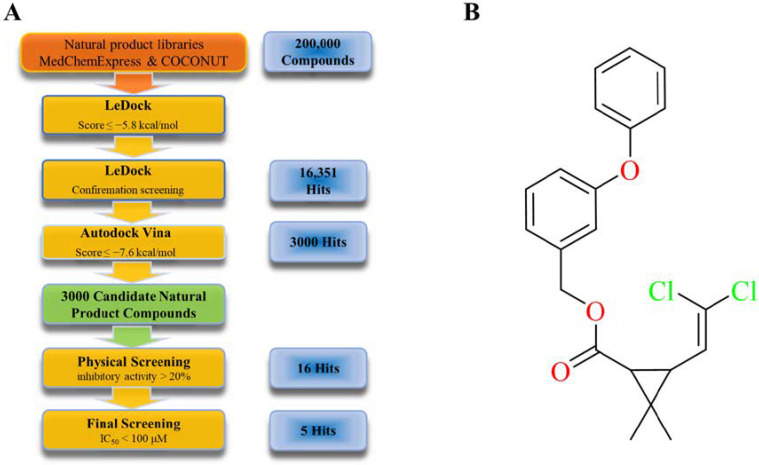
Screening process and chemical structure. (**A**) A guide to the entire screening process, including virtual and physical screening. (**B**) Chemical structure of permethrin.

This work performed a bulk virtual screening of furin inhibitors using LeDock [29] on natural product libraries containing approximately 200,000 compounds. Compounds scoring less than −5.8 kcal/mol were selected. After eliminating false positive results, 16,351 hit compounds were selected for the next round. For the second round of virtual screening, a refined docking was performed using AutoDock Vina [30,31] to select compounds with a Vina score of less than −7.6 kcal/mol, a commercial library including 3000 natural product compounds was selected for the physical screening experiment, and 16 compounds with inhibitory activity of more than 20% on furin were finally obtained. In order to ensure the accuracy of the screening results, those compounds were rescreened and tested three times, and their IC_50_ values were measured (Figure 1A, Table 1); we can see that there are five compounds with IC_50_ values within 100 μM. Among them, YJJ-B0887 is permethrin with an IC_50_ 38.72 μM and 55.89% inhibition at 50 μM. Based on the inhibitory activity combined with structural analysis, this research identified permethrin as the primary object of this study (Figure 1B).

To further verify whether permethrin also has inhibitory activity against other related proteases, TMPRSS2 and trypsin [32,33,34] proteases, which are also important in viral infections were used as controls, and the results showed that to neither enzyme, permethrin has inhibitory activity (Figure S1E), therefore it exhibits some protease selective inhibition.

### 2.3. Kinetic Characterization of Furin

**Figure 2:**
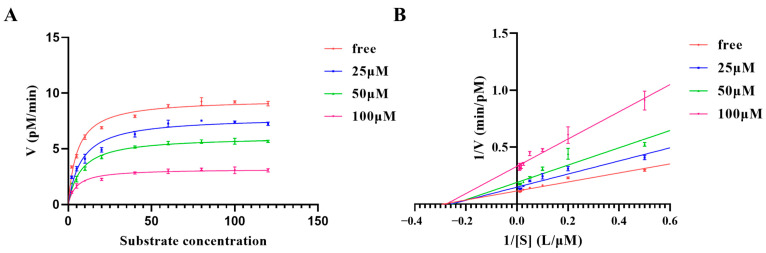
Michaelis–Menten inhibitory kinetics results of permethrin for furin. (**A**) Plot of substrate concentration against enzymatic reaction speed for permethrin concentrations of 0 μM, 25 μM, 50 μM, and 100 μM. (**B**) The inhibition curves in a Lineweaver–Burk plot at different permethrin concentrations with an average *x*-axis intercept of −0.3176.

Measurement of kinetic characterization contributes to a deeper understanding of the catalytic properties of furin and the mechanism of inhibition of permethrin. The results are shown in Figure 2; we can clearly observe that the maximum reaction rate of furin decreases with the addition of permethrin, while *K_m_* does not change. This phenomenon becomes apparent with increasing concentrations of permethrin (Figure 2A). The inhibition curves in a Lineweaver–Burk plot for different concentrations of permethrin intersected at the same position on the *x*-axis with almost the same intercept (−1/*K_m_*) (Figure 2B), indicating that changes in inhibitor concentration did not result in changes in the *K_m_* of furin. Such results allow us to hypothesize that permethrin might not bound to the classical catalytic pocket of furin and play a competitive inhibitory role; instead, it is likely to interact with furin in a novel binding mode, acting as a non-competitive inhibitor.

### 2.4. Interaction Mode of Permethrin with Furin

In order to investigate this new interaction mode of permethrin with furin, allosteric site prediction, molecular docking, interaction energy calculation, and molecular dynamics simulation were utilized in the study. These results will help us to better understand the mechanism of the interaction of permethrin with furin, as well as be used to guide the structural modification of permethrin in future work.

**Figure 3:**
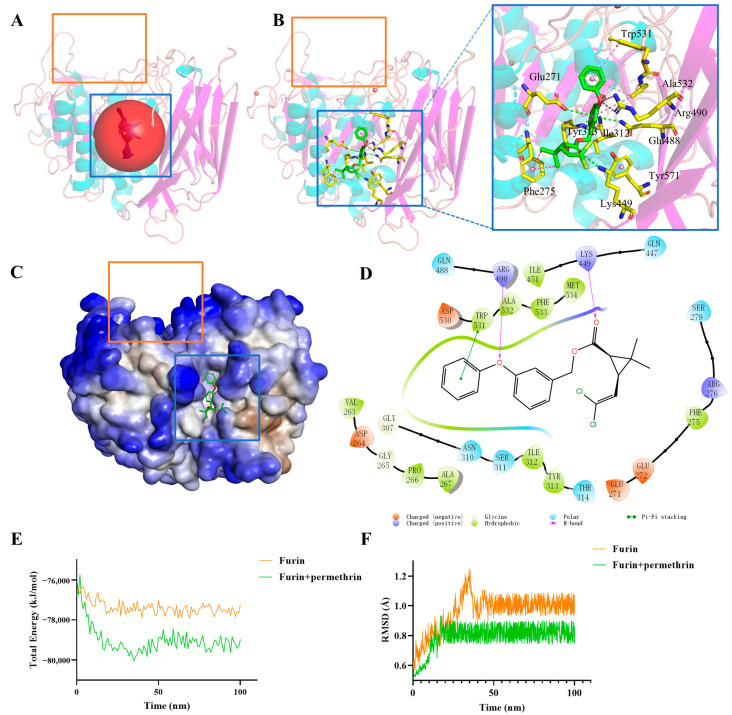
Interaction analysis of the permethrin–FuAP1 complex. (**A**) Red sphere represents allosteric site predicted by the AlloSitePro program. The blue and orange boxes represent the allosteric pocket and the catalytic pocket, respectively. (**B**) Furin interacts with permethrin. Cyan and purple represent the α-helix and β-sheet of furin, respectively; green represents permethrin; yellow represents the surrounding amino acid residues most related to permethrin action; red, blue, and light green represent oxygen, nitrogen, and chlorine, respectively; the pink and green dashed lines represent the interaction bonds of furin with permethrin. (**C**) Furin’s surface presentation format. Blue and red represent hydrophilic and hydrophobic sites, respectively. (**D**) A 2D display of the interaction between furin and permethrin. The triangles represent the amino acid residues around permethrin and are labeled red (negatively charged), purple (positively charged), green (hydrophobic), and blue (polar) according to their side chain chemical properties; the pocket is represented by a colored band between residues and permethrin; the pink arrow segments indicate H-bond forces, and the green dot segment signifies Pi–Pi stacking force. (**E**) Total simulated energy change of furin and the permethrin–FuAP1 complex using MD simulation. (**F**) RMSD analysis of furin and the permethrin–FuAP1 complex using MD simulation.

This study first used the allosteric site prediction program and found that the red sphere in Figure 3A is most likely its allosteric regulatory pocket, which we named furin allosteric pocket 1 (FuAP1) in this research. It is marked with a blue box in Figure 3 and Figure S2, while for comparison, we denoted the catalytic pocket as furin catalytic pocket (FuCP), marked with an orange box in Figure 3 and Figure S2. FuAP1 is located between the peptidase S8 domain (aa121-435) and the P/Homo B domain (aa444-576) of furin, forming a relatively independent hydrophobic pocket; this may be the structural basis for its capability of interacting with other molecules as a separate part. Equally important, this study also found that the FuAP1 pocket is always present whether furin is in the on or off state.

This work docked permethrin into furin’s FuAP1 and FuCP, respectively. As shown in Figure 3 and Figure S2 permethrin could also bind to FuCP; however, comparing the interaction energy of “permethrin–furin” (Table 2 and Table S1), the −49.60 kcal/mol of “permethrin–FuAP1” is much better than that of “permethrin–FuCP” (−13.29 kcal/mol). Among them, Lys449, Arg490, Glu271, and Ile312 contributed the most interaction energy, of respectively −25.80 kcal/mol, −14.82 kcal/mol, −5.36 kcal/mol, and −4.05 kcal/mol. At the same time, this research analyzed the interaction conformation (Figure 3B–D) showing that permethrin forms a more stable mode of action with FuAP1, such that Glu271 and Gln488 form a remote hydrogen bond through the benzene ring in the middle of permethrin, the amino group on Lys449 forms a hydrogen bond with the carbonyl group in permethrin, the guanidine group of Arg490 forms two hydrogen bonds with the oxygen atom, and Phe275, Ile312, Try313, Trp531, and Ala532 form hydrophobic effects with permethrin hydrophobic moieties. In contrast, the interaction between permethrin and FuCP was only Leu227, Val231, Trp254 forming three hydrophobic bonds with permethrin (Figure S2A–C). These findings all indicate that the primary interaction of permethrin with furin occurs in the FuAP1 pocket, rather than the FuCP pocket as traditionally perceived, which is also consistent with the enzymatic kinetic results indicating that permethrin should be bound to an allosteric regulatory pocket to inhibit the activity of furin in a non-competitive inhibition manner.

### 2.5. Molecular Dynamics Simulation

To further verify the interaction pattern of permethrin with furin, MD simulations of 100 ns duration were performed for furin, “permethrin–FuAP1 complex” and “permethrin–FuCP complex”. By comparing the MD simulation data of them, this study found that the total energy of “permethrin–FuAP1 complex” was lower than that of furin protein alone (Figure 3E), and this difference was not obvious in the simulation of “permethrin–FuCP complex”. A similar phenomenon was observed in the RMSD results; the structural stability of the “permethrin–FuAP1 complex” was significantly better than that furin itself (Figure 3F), and the average RMSD of the “permethrin–FuAP1”, was 0.793607, which was lower than that of furin itself at 0.958682, while the RMSD of the “permethrin–FuCP complex” was not significantly different from that of furin itself (Figure S2E). These MD simulation data suggest that permethrin has a better binding capacity in FuAP1 than in FuCP and stabilizes the whole complex.

### 2.6. Cellular Experiment Results

**Figure 4:**
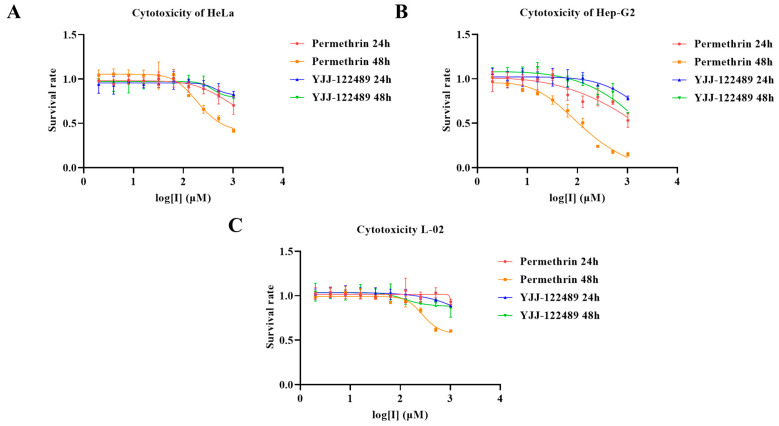
Cellular cytotoxicity of permethrin and YJJ-122489. (**A**) Logarithm of molecule concentration against HeLa cell viability. (**B**) Logarithm of molecule concentration against Hep-G2 cell viability. (**C**) Logarithm of molecule concentration against L-02 cell viability.

To verify that the inhibitory ability of permethrin on furin is not only reflected at the molecular level, but also effective at the cellular level in this work we performed cytological studies using tumor cell lines strongly expressing furin (Hep-G2 and HeLa) and a control cell line (L-02). YJJ-122489, which had shown no inhibitory activity against furin in the previous drug screening, was also used as a control molecule. The cytotoxicity with increasing concentration of permethrin and YJJ-122489 was assayed by incubating cells treated with a gradient of compound concentration for 24 or 48 h (Figure 4). The results showed that permethrin had a stronger cytotoxic effect on the liver tumor cell line Hep-G2, and a higher cytotoxic effect on a non-hepatic tumor cell line (HeLa) also with a high furin expression, while the killing of normal liver cells L-02 with low furin expression was much weaker than that of Hep-G2 cancer cells from the same tissue. As a control molecule, YJJ-122489 had no significant cytotoxic effect on all three cell lines. The results showed that permethrin was more potent against tumor cells, especially furin-expressing tumor model cell lines, while it showed weaker toxicity against the corresponding non-tumor cell lines. Such results suggest that permethrin does not only exhibit furin inhibitory activity at the molecular level, but it still has the ability to inhibit furin and related functions at the cellular level.

## 3. Discussion

In this study, although the *Pyrethrum cinerariifolium* Trev. ingredient derivative permethrin has obvious inhibitory activity against furin, it is still a long way from being a drug, and we will use it as a lead compound for structural modification and optimization to acquire derivatives with higher inhibitory activity and more stable structure in our future research. In Figure 3, the non-polar group of permethrin has a strong affinity to the hydrophobic residues of furin, forming five groups of hydrophobic interactions and contributing strongly to the binding energy. The chlorine atoms, however, do not contribute much to the binding forces and do not show a significant bonding effect. The non-polar group has also been reported to be an important source of toxicity for permethrin [35,36]. Therefore, in our future work we will first replace this part with a carbonyl-like group that can form good conformational complementation and interaction with Arg276 and Ser279 sites.

Most of the existing furin inhibitors were developed based on furin-substrate peptide analogs, which have good binding to the catalytic pocket of furin but lack chemical and structural diversity [18,37]. Additionally, given the importance of furin in multiple diseases including SARS-CoV-2 infection and tumors, its biochemical studies need to be more advanced [7,8,38,39]. In the present work, a potential inhibitor of furin with a novel backbone was identified, more importantly, its novel non-competitive allosteric regulatory mode was discovered through enzyme kinetic experiments and structural simulation studies. However, due to the limitations of the experimental scale, this study did not fully elucidate the inhibition of furin by permethrin and its mechanism at the in vivo level, and also lacked validation assays related to anti-SARS-CoV-2 infection. In our future work, we will conduct functional studies on optimized active permethrin derivatives to verify and investigate the overall effects and molecular mechanisms of permethrin and its derivatives on furin inhibition in anti-tumor and anti-SARS-CoV-2 infections. In turn, new strategies and drug candidates will be provided for the prevention and treatment of COVID-19 and some cancers.

## 4. Materials and Methods

### 4.1. Expression and Purification of Furin

The commercial expression pET30a vector containing the furin gene (GenScript, Nanjing) was transduced into *E. coli* BL21 (DE3). After resuscitation of the transformed cells in LB medium containing kanamycin (100 mg/L) at 37 °C for 12 h, the cells were expanded at 37 °C (200 rpm) for 3 h until OD600 reached 0.6. Expression was induced with 0.1 mM IPTG, and the cells were harvested at 14 °C (200 rpm) after 16 h. Cells were collected using 6000 rpm for 10 min and resuspended in 50 mL of 25 mM Tris buffer (pH 7.5, containing 150 mM NaCl, 1 mM CaCl_2_). The cell suspension was sonicated (power setting 200 W) for 20 min and centrifugation at 12,000 rpm for 10 min. The precipitate with furin was collected, washed with 50 mM Tris buffer (pH 7.5, containing 1 M NaCl, 1 mM CaCl_2_, 2 M urea, 1% Triton X-100), then dissolved in 50 mM Tris buffer (pH 7.5, containing 150 mM NaCl, 1 mM CaCl_2_, 8 M urea, and 2 mM DTT), filtered using a 0.22 μM microporous membrane (Acrodisc, Puerto Rico), and subsequently used for chromatographic purification using a Ni-NTA column (Qiagen, Germany). After SDS–PAGE analysis to determine the effluent fraction containing the target protein, we used 50 mM Tris buffer (pH 7.5, containing 150 mM NaCl, 1 mM CaCl_2_, 10% glycerol, 1 M L-Arg and 2 mM DTT) in a dialysis liquid system for 12 h at 4 °C, then the protein solution was loaded into a gravity desalination column (Bio-Rad, USA) which was pre-balanced using 100 mM HEPES buffer (pH 6.0, containing 5 mM CaCl_2_). Then it was mixed with 10% glycerol and stored at −80 °C until use. Finally, the furin was identified using SDS–PAGE and Western blot.

### 4.2. Virtual Inhibitor Screening and Molecular Dynamics Simulation

In this project, the natural product libraries of Med Chem Expression and COCONUT [40], and the crystal structure of furin (PDB ID: 4Z2A) were used for virtual screening. Firstly, LeDock was employed as a rapid screening tool for the initial screening of 200,000 compounds, the compounds with LeDock scores of less than −5.8 kcal/mol were filtered, and they were screened repeatedly to rule out unstable and false-positive results. Afterwards, AutoDock Vina was performed for fine docking and 3000 selected natural product molecules with Vina results of less than −7.6 kcal/mol were ultimately procured for physical screening experiments. All compounds used for physical screening were sourced from MCE and through experiments and calculations, several candidate compounds were identified. The potential inhibitor with most important effect was selected: permethrin. Molecular dynamics simulations were performed for 100 ns using the SPC216 model of the G43A1 force field with the addition of 3D water box (3.3 × 3.3 × 2.3 nm) and 140 mM concentrations of Na^+^ and Cl^−^, using GROMACS [41] software. In addition, PyMOL was utilized throughout the article for the visualization of the 3D structure.

### 4.3. Furin Activity Assay and Enzymatic Kinetics

Boc-Arg-Val-Arg-Arg-AMC (Boc-R-V-R-R-AMC) was used as a substrate for the detection of furin. It carries an AMC (7-methyl coumarin) moiety that fluoresces at an excitation wavelength of 370 nm and an emission wavelength of 460 nm.

Inhibitor screening was performed using 96-well plates with a 200 μL reaction system per well consisting of furin in 100 mM HEPES buffer (pH 6.0, containing 0.5% Triton X-100 and 5 mM CaCl_2_) pre-incubated with the different inhibitors to be tested at 37 °C for 5 min. The reaction was then incubated for 90 min at 37 °C, supplemented with 40 μM substrate, quenched using 1% formic acid and the enzyme activity was monitored using a Thermo Scientific Varioskan Flash Multi-Mode Microplate Reader.

For the measurement of enzymatic kinetics and inhibition kinetics, the substrate concentration gradients were set to 0 μM, 2 μM, 5 μM, 10 μM, 20 μM, 40 μM, 60 μM, 80 μM, 100 μM, 120 μM, and the compound concentration gradients to 0 μM, 25 μM, 50 μM, 100 μM. Furin was added to 100 mM HEPES assay system buffer (pH 6.0, containing 0.5% Triton X-100 and 5 mM CaCl_2_), followed by different concentrations of the compounds to be tested, preincubated at 37 °C for 5 min. Different concentrations of the substrate were added; the sample was incubated at 37 °C for 35 min, and quenched with 1% formic acid. The initial rate of the enzymatic reaction of furin was tested and the curve was plotted.

### 4.4. Allosteric Site Prediction

Based on the results of the enzymatic kinetics and structural calculation, this study found that the inhibitory model of permethrin was not a classical competitive inhibition, and this work speculated that it probably acted on another allosteric site. Via AlloSitePro (http://mdl.shsmu.edu.cn/AST/, accessed on 14 December 2022) was used to analyze the different state conformations of furin (PDB ID: 5 JXG, 5 JXJ), and in consideration of the studies by Sven O. Dahms et al. [20,21,22], this research confirmed the existence of other compound binding sites for furin that could influence the catalytic efficiency of the protein by changing the state of furin.

### 4.5. Cellular Experiments

Furin is highly expressed in liver cancer cells and has specific effects on hepatocellular carcinoma. Therefore, human hepatocellular carcinoma cells Hep-G2 were selected as experimental cells, cervical carcinoma cells HeLa with similar furin expression in Hep-G2 were selected as control, and human permanent hepatocytes L-02 were selected as negative control. DMEM was used for HeLa, and 1640 medium was used for Hep-G2 and L-02. After incubation for 24 h with 100 μL complete medium in 96-well plates with 8000 cells per well, the medium was aspirated, 100 μL serum-free medium containing a concentration gradient of the compound was added and the samples were incubated. After 24 or 48 h the drug-containing medium was aspirated and 100 μL medium solution containing 10% CCK8 was added, subsequently, the absorbance was measured at 450 nm using a microplate reader after 1 h of incubation.

## 5. Conclusions

In this study, furin with high purity and activity was obtained using recombinant expression, and the activity detection system was optimized. Next, a virtual screening of a natural product library containing 200,000 natural product molecules and a target-based physical screening at the enzyme level were combined and an effective potential inhibitor against furin, permethrin, was identified. The most important contribution of this investigation is the demonstration of a novel binding mode of permethrin to furin, and the discovery of this non-competitive inhibition phenomenon will contribute to a better understanding of the biochemical characteristics of furin and to the design of novel therapeutic drugs based on it. In addition, this study also demonstrated the good selective inhibitory ability of the pyrethrin I derivative permethrin against furin, as well as the inhibitory ability and anti-tumor-cell-proliferation activity of permethrin against furin at the cellular level. These findings may provide new insights and lead compounds for the discovery of drugs against furin.

## Figures and Tables

**Table 1:** Inhibitory effects of small molecule inhibitors obtained by screening.

Compound	IC_50_ (μM)	Inhibition Rate (50 μM)
YJJ-N2376	83.36	29.41%
YJJ-N3443	75.82	33.49%
YJJ-N0522	73.78	43.31%
YJJ-B0887	38.72	55.89%
YJJ-18963	46.97	48.64%
YJJ-N0120	101.83	25.61%
YJJ-N0290	112.54	22.13%
YJJ-14855B	128.33	25.04%
YJJ-N4184	134.62	27.28%
YJJ-N2350	141.99	26.45%
YJJ-N0594	102.81	31.00%
YJJ-N0824	113.56	23.56%
YJJ-N1478	130.16	27.18%
YJJ-N1967	142.36	25.87%
YJJ-B2176A	123.76	19.31%
YJJ-122489	145.27	21.24%

**Table 2:** Calculation of the interaction energy between FuAP1 and permethrin.

Residue	Interaction Energy (kcal/mol)	VDW Interaction Energy (kcal/mol)	Electrostatic Interaction Energy (kcal/mol)
Glu271	−5.359410	−3.310534	−2.048877
Phe275	−0.127889	−2.632041	2.504151
Ile312	−4.050157	−1.933321	−2.116836
Tyr313	0.804807	−4.178134	4.982942
Lys449	−25.798443	−2.144799	−23.653643
Gln488	−1.351767	−2.007794	0.656026
Arg490	−14.920117	−2.468614	−12.451504
Trp531	0.748106	−1.649797	2.397903
Ala532	−1.503742	−1.748421	0.244680
Tyr571	1.961132	−1.538561	3.499692
Total	−49.59748	−23.61202	−25.98546

## Data Availability

The data presented in this study are available on request from the corresponding authors.

## Supplementary Materials

The following supporting information can be downloaded at: https://www.mdpi.com/article/10.3390/molecules28041883/s1, Figure S1: Results of expression, purification, and activity assays of furin; Table S1: Calculation of the interaction energy between FuCP and permethrin; Figure S2: Interaction analysis of the permethrin–FuCP complex.
