# Supplementary material for: Permethrin as a Potential Furin Inhibitor through a Novel Non-Competitive Allosteric Inhibition
Source: Molecules. 2023 Feb 16;28(4):1883. doi: 10.3390/molecules28041883 (PMC9959265; doi:10.3390/molecules28041883)
Supplement: Supplementary file 1 [file molecules-28-01883-s001.zip › molecules-2214145-supplementary.pdf]

molecules

Permethrin as a Potential Furin Inhibitor through a Novel Non-Competitive Allosteric Inhibition

Supplementary materials

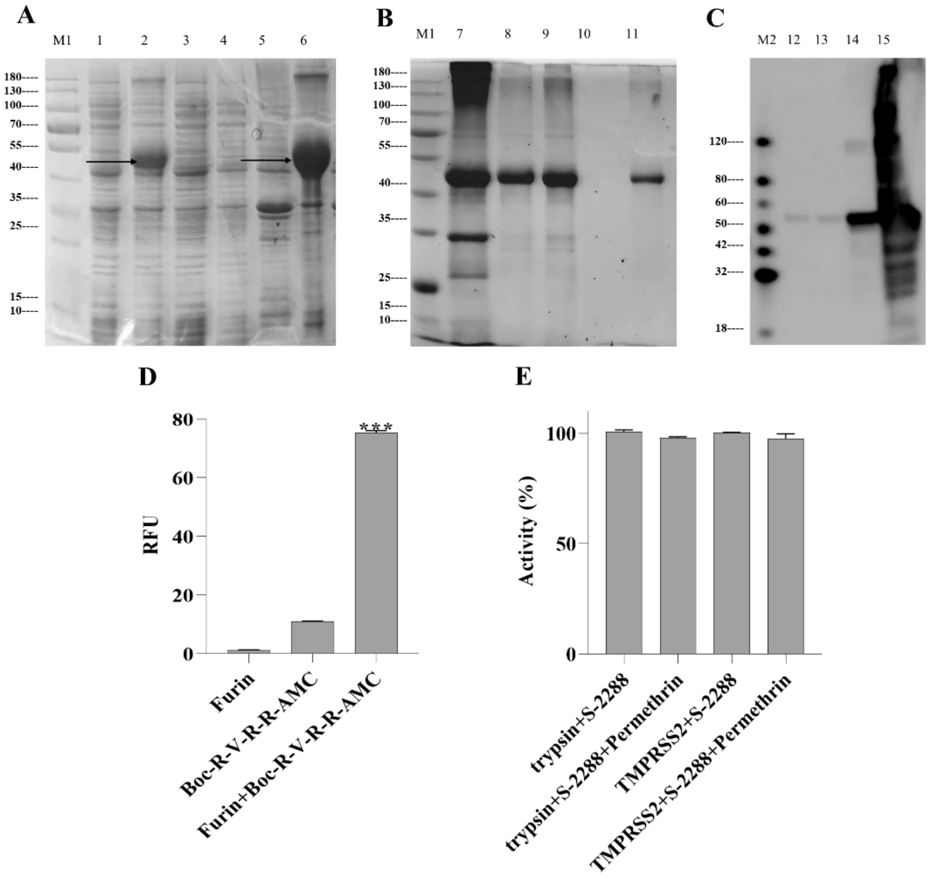

Figure S1. Results of expression, purification and activity assay of furin. (**A**) Expression results of furin protein and identification by SDS-PAGE. M1: Protein Marker; 1: Total protein not induced; 2: Total protein induced: 3: Uninduced supernatant; 4: Induced supernatant; 5: Uninduced precipitation; 6: Induced precipitation. (**B**) Ni-NTA column affinity chromatography purification results of furin analyzed by SDS-PAGE, M1: Protein Marker; 7: Induced precipitation total protein; 8: Load2; 9: Load3; 10: Wash; 11: Elute. (**C**) Western-blot analysis of furin expression results, M2: Western blot Marker; 12: Supernatant of cell lysate with induction for 16 h at 14 °C; 13: Supernatant of cell lysate with induction for 5 h at 37 °C; 14: Precipitation of cell lysate with induction for 16h at 14 °C; 15: Precipitation of cell lysate with induction for 5 h at 37 °C. The primary antibody for western blot is anti-His antibody (GenScript, Cat.No.A00186) (**D**) Furin activity determination; $P < 0.001$. (**E**) Inhibition effect of permethrin on trypsin and TMPRSS2.

Table S1. Calculation on the interaction energy between FuCP with permethrin.

Residue	Interaction Energy (kcal/mol)	VDW Interaction Energy (kcal/mol)	Electrostatic Interaction Energy (kcal/mol)
His194	-4.506349	-2.798474	-1.707875
Leu227	-3.757178	-3.433364	-0.323814
Asn295	-5.027681	-2.378916	-2.648765
Total	-13.29121	-8.61075	-4.68045

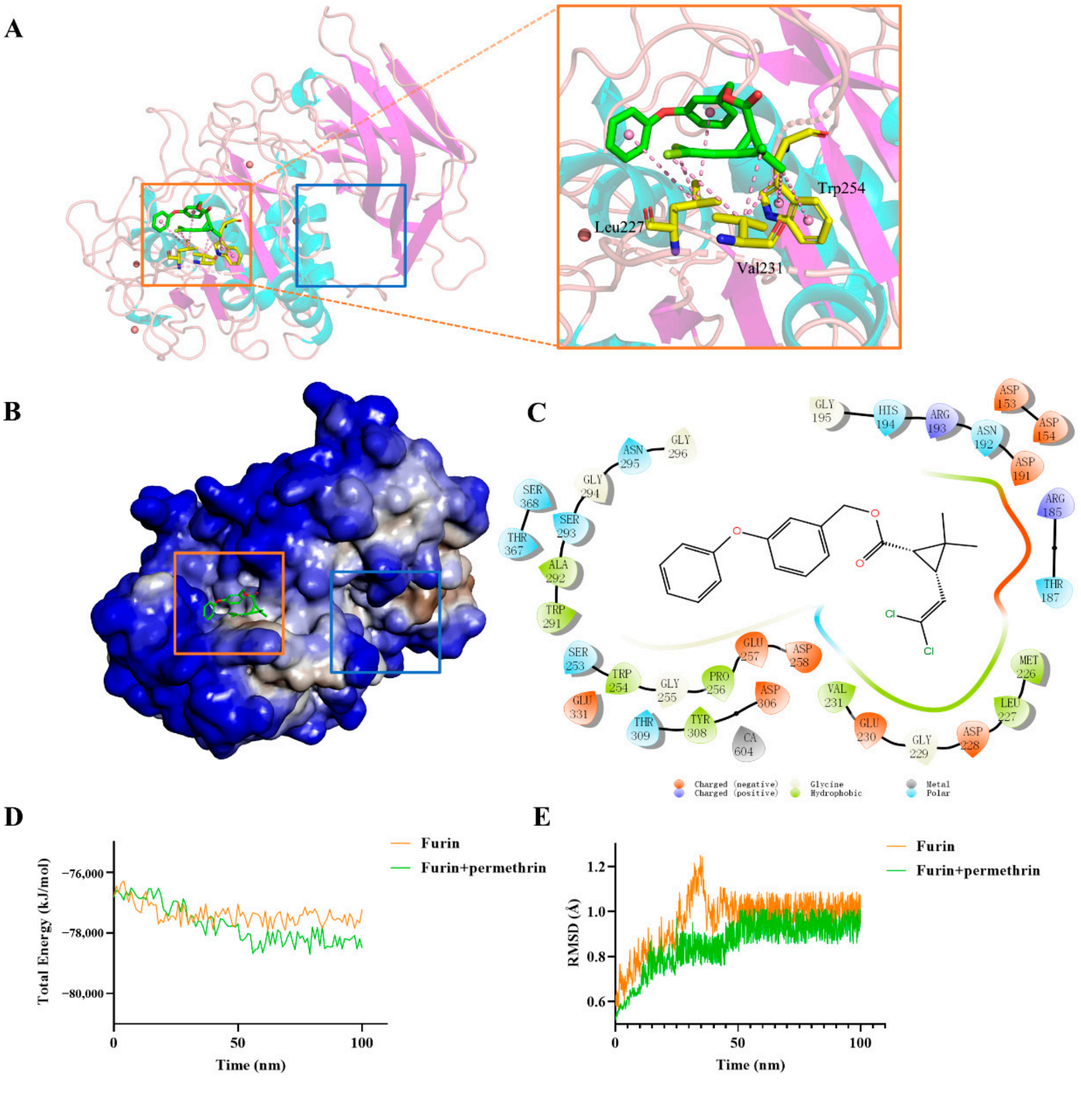

Figure S2. Interaction analysis of permethrin-FuCP complex. (**A**) FuCP interacts with permethrin, cyan and purple represent the α-helix and β-sheet of furin, green represents permethrin, yellow represents the surrounding amino acid residues most related to permethrin action, red, blue, light green represent oxygen, nitrogen, chlorine, the pink dashed lines represent the interaction bonds of furin with permethrin. The blue and orange boxes represent the allosteric pocket and the catalytic pocket, respectively. (**B**) Furin's surface presentation format. Blue and red represent hydrophilic and hydrophobic, respectively. (**C**) 2D display of the interaction between FuCP and permethrin. The triangles represent the amino acid residues around permethrin and are labeled red (negatively charged), purple (positively charged), green (hydrophobic), and blue (polar), respectively, according to their side chain chemical properties; the pocket is represented by a colored band between residues and permethrin. (**D**) Total energy change simulated of furin and permethrin-FuCP complex by MD simulation. (**E**) RMSD analysis of furin and permethrin-FuCP complex by MD simulation.
